# Direct oral anticoagulants versus warfarin for left ventricular thrombus: an updated systematic review and meta-analysis of randomized and observational studies

**DOI:** 10.3389/fcvm.2026.1814694

**Published:** 2026-05-14

**Authors:** Lama Alfehaid, Jinan Alhuwayshil, Shoug Almousa, Ehsan Habeeb, Majed S. Al Yami

**Affiliations:** 1 Department of Pharmacy Practice, College of Pharmacy, King Saud bin Abdulaziz University for Health Sciences, Riyadh, Saudi Arabia; 2 Pharmaceutical Care Department, King Abdulaziz Medical City, Riyadh, Saudi Arabia; 3 Department of Pharmacy Practice, King Abdullah International Medical Research Center, Riyadh, Saudi Arabia; 4 Department of Pharmacy Practice, College of Pharmacy, Taibah University, Madinah, Saudi Arabia

**Keywords:** bleeding, direct oral anticoagulants, left ventricular thrombus, meta-analysis, mortality, systemic embolism, warfarin

## Abstract

**Background:**

Left ventricular thrombus (LVT) is associated with substantial risk of embolism and mortality. Given the growing use of direct oral anticoagulants for LVT and evolving evidence, we conducted an updated, comprehensive systematic review and meta-analysis comparing their effectiveness and safety with vitamin K antagonists.

**Methods:**

We systematically searched PubMed/MEDLINE, Embase, and the Cochrane Central Register of Controlled Trials from inception through the most recent search date for randomized controlled trials and observational studies comparing DOACs with VKAs in adults with imaging-confirmed LVT. Outcomes of interest included LVT resolution, systemic embolic events, all-cause mortality, bleeding events, and composite clinical outcomes. Pooled risk ratios (RRs) with 95% confidence intervals (CIs) were calculated using random-effects models. Statistical heterogeneity was assessed using the I² statistic.

**Results:**

A total of 29 studies, comprising randomized controlled trials and observational cohorts, were included. Compared with VKAs, DOAC therapy was associated with a numerically higher likelihood of LVT resolution, although this did not reach statistical significance (RR 1.06, 95% CI 0.98–1.14; I² = 48.2%). There was no significant difference in the risk of systemic embolic events between DOACs and VKAs (RR 0.89, 95% CI 0.78–1.03; I² = 5.5%). DOAC use was associated with a lower risk of all-cause mortality (RR 0.84, 95% CI 0.64–1.09; I² = 5.5%); however, this finding was primarily driven by observational data. DOACs were also associated with a lower risk of bleeding events (RR 0.86, 95% CI 0.72–1.03; I² = 16.4%), although this did not reach statistical significance.

**Conclusions:**

In this updated meta-analysis, DOACs were associated with a numerically higher rate of LVT resolution, although this did not reach statistical significance, and with lower rates of bleeding compared with VKAs, without an increased risk of systemic embolic events. Although a lower risk of all-cause mortality was observed, this finding was primarily driven by observational studies and should be interpreted with caution. These findings support DOACs as a reasonable and potentially safer alternative to VKAs for the management of LVT, while emphasizing the need for adequately powered randomized trials to confirm optimal anticoagulation strategies in this population.

**Systematic Review Registration:**

https://www.crd.york.ac.uk/PROSPERO/view/CRD420251181379, PROSPERO CRD420251181379.

## Introduction

Left ventricular thrombus (LVT) typically develops most commonly after an acute myocardial infarction (MI), in chronic cardiomyopathy with a reduced ejection fraction, or in the context of hypercoagulable states. LVT typically forms within 1–14 days after MI and is associated with higher risks of systemic embolism (stroke/TIA/peripheral embolism), recurrent MI, and death, with many thromboembolic events clustering in the first four months post-MI. Even with anticoagulation, persistent or recurrent thrombus and embolic complications remain clinically significant (1–4).

Historically, vitamin K antagonists (VKAs), most commonly warfarin, have been the standard therapy for LVT with a target INR 2.0–3.0, and a typical treatment duration of 3–6 months adjusted to thrombus resolution, LV recovery, and bleeding risk. This approach is reflected in major guidance, as the American Heart Association (AHA) 2022 Scientific Statement on LVT recommends initiating therapeutic oral anticoagulation for post-MI LVT “typically for 3 months.” The statement also provides practical management suggestions for non-ischemic cardiomyopathy–related LVT (1, 5–7).

From the European perspective, the 2017 ESC STEMI Guideline recommended oral anticoagulation for at least 6 months when LVT is present, with imaging-guided continuation; the more recent 2023 ESC Acute Coronary Syndromes (ACS) Guideline continues to acknowledge anticoagulation for LVT within comprehensive ACS care pathways, with duration individualized and guided by follow-up imaging (4, 8, 9).

In 2025, the ACC/AHA ACS Guideline updated broader ACS management and recognized the evolving evidence around LVT; while VKAs remain the traditional standard (8), contemporary guidance and expert summaries increasingly note that either VKAs or a direct oral anticoagulants (DOACs) may be considered for ∼3–6 months in LVT, acknowledging that high-certainty, LVT-specific randomized evidence is limited (9).

Meanwhile, DOACs, including apixaban, rivaroxaban, dabigatran, and edoxaban, are firmly established in non-valvular atrial fibrillation and venous thromboembolism due to their predictable pharmacokinetics, rapid onset, fewer drug/food interactions, and the absence of routine INR monitoring. These advantages have prompted the growing use of DOACs for LVT. Early observational cohorts, as well as emerging randomized trials, suggest comparable effectiveness to VKAs for thrombus resolution and embolic prevention, although results remain heterogeneous across settings and designs.

Accordingly, this updated systematic review and meta-analysis will synthesize randomized and observational evidence comparing DOACs vs. VKAs for LVT, focusing on thrombus resolution, embolic events, bleeding, and mortality, to inform clinical decision-making and the next iterations of guideline recommendations.

## Methods

This systematic review and meta-analysis was conducted and reported in accordance with the Preferred Reporting Items for Systematic Reviews and Meta-Analyses (PRISMA) 2020 statement (10), and the study protocol was registered in PROSPERO (CRD420251181379). The completed PRISMA 2020 checklist is provided in Supplementary Table S1.

### Study design and eligibility criteria

We systematically identified randomized and observational studies evaluating the comparative effectiveness and safety of DOACs vs. VKAs in patients with LVT. Eligible studies enrolled adult patients (≥18 years) with LVT confirmed by imaging, including transthoracic or contrast echocardiography, cardiac magnetic resonance imaging, or computed tomography, irrespective of the underlying etiology, such as post–myocardial infarction, non-ischemic cardiomyopathy, or other causes.

Studies were required to evaluate treatment with a DOACs (apixaban, rivaroxaban, dabigatran, or edoxaban) and include a comparator group receiving warfarin or another VKAs. To be eligible, studies had to report at least one clinically relevant outcome, including thrombus resolution on follow-up imaging, systemic embolic events, all-cause mortality, and bleeding events. Randomized controlled trials, prospective or retrospective cohort studies, and case–control studies were included. Case reports, small case series, review articles, editorials, conference abstracts without full data, and non-peer-reviewed literature were excluded.

### Data sources and search strategy

A comprehensive literature search was performed in PubMed/MEDLINE, Embase, and the Cochrane Central Register of Controlled Trials from database inception through the most recent search date. The search strategy combined controlled vocabulary terms and free-text keywords related to LVT, direct oral anticoagulants, warfarin, and individual DOAC agents; the complete search strategies for PubMed/MEDLINE, Embase, and Cochrane CENTRAL are provided in the Supplementary Materials (Supplementary Table S2). No restrictions were applied based on year of publication. Only studies published in English were considered.

To ensure completeness, reference lists of included studies and relevant reviews were manually screened. Additional searches were conducted using Google Scholar and selected high-impact cardiovascular and thrombosis journals. The complete electronic search strategy is provided in the Supplementary Appendix S1.

### Study selection

All identified records were imported into a reference management system, and duplicate citations were removed. Two reviewers independently screened titles and abstracts to identify potentially eligible studies. Full texts of relevant articles were then reviewed in detail to determine final inclusion. Any disagreements were resolved through discussion, and when necessary, by consultation with a third reviewer. The study selection process is summarized in a PRISMA 2020 flow diagram.

### Data extraction

Data extraction was performed independently by two reviewers using a standardized, pilot-tested data collection form. Extracted variables included study characteristics (first author, publication year, country, study design, and setting), patient demographics and LVT etiology, intervention details (type, dose, and duration of DOAC therapy), comparator characteristics (VKA regimen and target INR), reported clinical outcomes, and duration of follow-up.

Where available, additional details on DOAC type, dosing regimens, and treatment duration—including continuation after thrombus resolution were collected. However, reporting of dose-specific outcomes and post-resolution anticoagulation duration was inconsistent across studies, limiting further stratified analyses.

Any discrepancies in data extraction were resolved through consensus, with arbitration by a third reviewer when necessary. The final dataset was cross-checked to ensure accuracy and completeness prior to analysis.

### Risk of bias assessment

The methodological quality of included studies was assessed independently by two reviewers according to study design. Randomized controlled trials were evaluated using the Cochrane Risk of Bias tool version RoB 2.0 (11), while observational studies were assessed using the ROBINS-I tool (12). Each study was categorized as having low, moderate (some concerns), or high risk of bias. Disagreements were resolved through discussion or adjudication by a third reviewer. Risk-of-bias assessments were incorporated into the interpretation of findings and into prespecified sensitivity analyses.

### Data synthesis and statistical analysis

A qualitative narrative synthesis was conducted for all included studies. Quantitative synthesis was performed when at least two studies reported the same outcome. Given the limited number and size of randomized trials in this field, randomized and observational studies were pooled using random-effects models to provide an overall estimate of treatment effects, while accounting for anticipated clinical and methodological heterogeneity. Prespecified subgroup and sensitivity analyses were performed according to the study design to explore the robustness of the pooled estimates.

Dichotomous outcomes were summarized using risk ratios or odds ratios with corresponding 95% confidence intervals, while time-to-event outcomes were summarized using hazard ratios when available. Statistical heterogeneity was assessed using the Cochran *Q* test and quantified using the I² statistic, with I² values greater than 50% indicating substantial heterogeneity.

### Subgroup and sensitivity analyses

Prespecified subgroup analyses were conducted based on study design (randomized controlled trials vs. observational studies). Sensitivity analyses were performed by excluding studies at high risk of bias and studies with disproportionate influence on pooled estimates. Publication bias was assessed using visual inspection of funnel plots and Egger's regression test, with results presented in the Supplementary Materials (Figures S1–S4).

## Results

### Study selection

The systematic literature search identified 576 records, including 191 from PubMed/MEDLINE, 50 from the Cochrane Central Register of Controlled Trials, and 68 from Embase. After removing 318 duplicate records, 258 unique records were screened by title and abstract. Of these, 227 records were excluded during initial screening as irrelevant to the study question.

**Figure 1:**
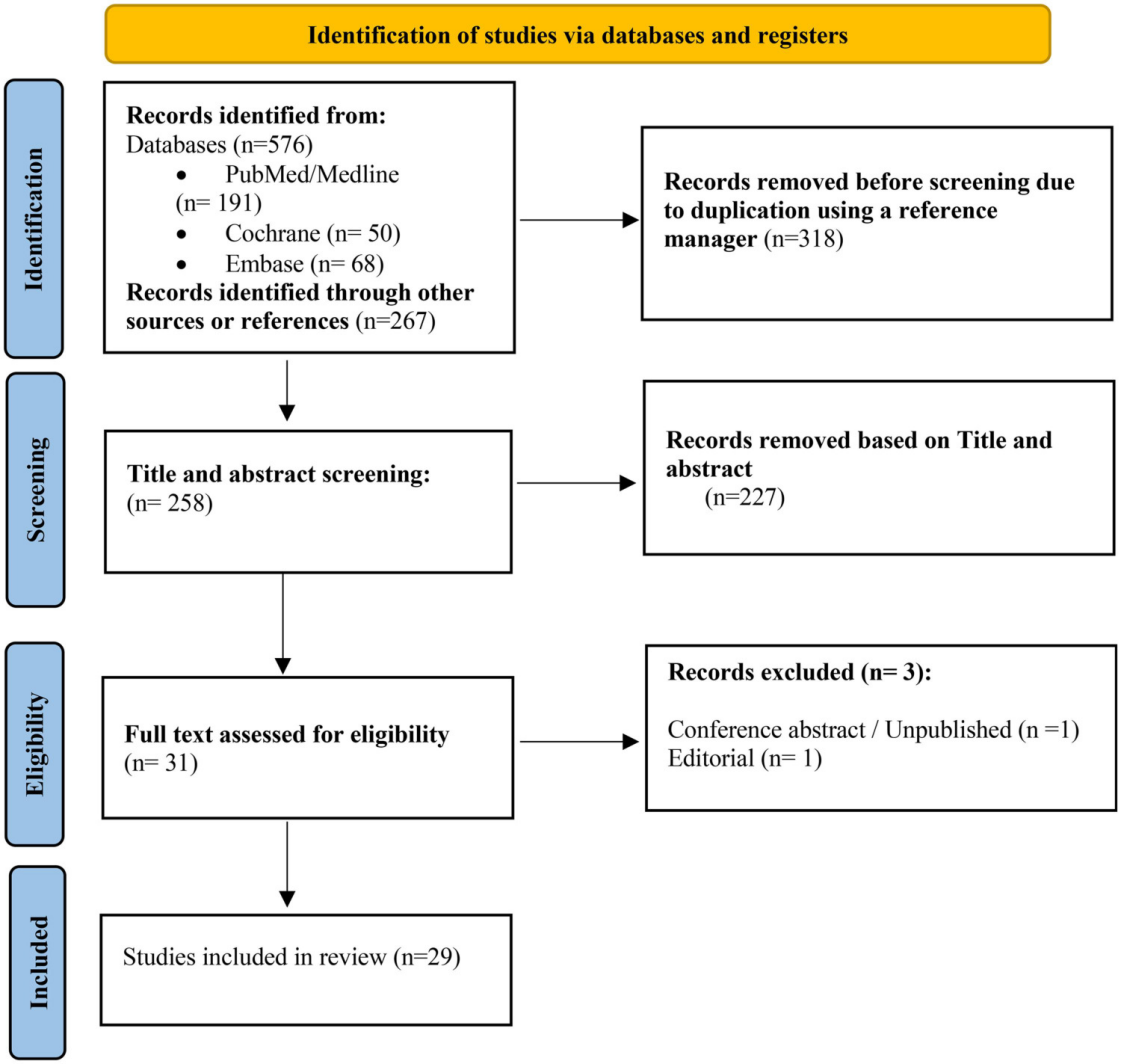
PRISMA 2020 flow diagram illustrating study selection. A total of 29 studies were included, comprising 7 randomized controlled trials, 1 prospective observational cohort study, and 21 retrospective observational cohort studies.

A total of 31 full-text articles were assessed for eligibility. Three studies were subsequently excluded at the full-text stage: one conference abstract without complete data and two editorials. This left 29 studies that met the predefined inclusion criteria and were included in the qualitative synthesis and meta-analysis. The study selection process is summarized in the PRISMA 2020 flow diagram (Figure 1).

### Characteristics of included studies

A total of 29 studies were included, comprising 7 randomized controlled trials, 1 prospective observational cohort study, and 21 retrospective observational cohort studies, with a combined sample of 5,229 patients with LVT. Of these, 2,074 patients received DOACs and 3,155 received VKAs, as summarized in Table 1.

**Table 1:** Summary of studies included (*n* = 29).

Study (Year)	Design	N	DOAC/VKA	Age	Male (%)	LVT Etiology	Imaging	DOAC	DOAC Dose	Antiplatelet Regimen	Follow-up	Primary Outcome
Shah (13)	Randomized controlled trial	261	171/90	54.5	79	Post–myocardial infarction	Transthoracic echocardiography	Rivaroxaban	20 mg once daily	Triple therapy → single antiplatelet therapy (clopidogrel)	12 weeks	LVT resolution
Mansouri (3)	Randomized controlled trial	52	26/26	56.5	85	Acute coronary syndrome	Transthoracic echocardiography	Rivaroxaban	20 mg once daily	Triple therapy → dual therapy (DOAC + clopidogrel)	3 months	LVT resolution
Youssef (14)	Randomized controlled trial	50	25/25	52.0	88	Post–myocardial infarction	Transthoracic echocardiography	Apixaban	5 mg twice daily	Triple therapy → single antiplatelet therapy	6 months	LVT resolution
Jenab (15)	Randomized controlled trial	50	26/24	55	82	ST-elevation myocardial infarction	Transthoracic echocardiography	Rivaroxaban	20 mg once daily	Dual antiplatelet therapy	3 months	LVT resolution
Ali (16)	Randomized controlled trial	35	18/17	57.1	80	Post–myocardial infarction	Transthoracic echocardiography	Apixaban	5 mg twice daily	Triple therapy → single antiplatelet therapy	3 months	LVT resolution
Abdelnabi (17)	Randomized controlled trial	79	39/40	49.6	57	Mixed	Transthoracic echocardiography	Rivaroxaban	20 mg once daily	Dual antiplatelet therapy (∼53%)	6 months	LVT resolution
Isa (18)	Randomized controlled trial	27	14/13	55.2	93	Mixed	Transthoracic echocardiography	Apixaban	5 mg twice daily (dose reduction applied)	Not reported	12 weeks	LVT resolution
Ali (16)	Retrospective cohort	110	32/60	59	79	Mixed	Echocardiography/cardiac magnetic resonance	Multiple DOACs	Standard doses	Single antiplatelet therapy predominant	1 year	Stroke/systemic embolism
Robinson (19)	Retrospective cohort	514	185/300	58.4	74	Mixed	Transthoracic echocardiography	Multiple DOACs	Standard doses	Not reported	351 days	Stroke/systemic embolism
Iqbal (20)	Retrospective cohort	84	22/62	62	89	Mixed	Echocardiography/cardiac magnetic resonance	Multiple DOACs (rivaroxaban, apixaban, dabigatran)	Standard doses	Single antiplatelet therapy 65%; dual antiplatelet therapy 38%	3 years	Thromboembolism
Guddeti (21)	Retrospective cohort	99	19/80	61	71	Mixed	Transesophageal echocardiography	Multiple DOACs	Standard doses	Triple therapy reported in subset	10 months	Stroke/bleeding
Daher (22)	Retrospective cohort	59	17/42	62	83	Post–myocardial infarction	Transesophageal echocardiography	Apixaban, dabigatran, rivaroxaban	Dose adjusted	Aspirin 58.8%; P2Y12 inhibitor 64.7%	3 months	LVT resolution
Xu (23)	Retrospective cohort	87	25/62	61.5	76	Mixed	Transesophageal echocardiography	Rivaroxaban or dabigatran	Standard doses	Not reported	2.4 years	Stroke/systemic embolism
Mihm (24)	Retrospective cohort	108	33/75	62	71	Post–myocardial infarction	Echocardiography/cardiac magnetic resonance	Apixaban or rivaroxaban	Standard doses	Aspirin 57.6%; P2Y12 inhibitor 21.2%	6 months	Stroke/systemic embolism
Jones (25)	Prospective cohort	101	41/60	Not reported	Not reported	Post–myocardial infarction	Transthoracic echocardiography	Apixaban or rivaroxaban	Standard doses	Triple therapy 68%; dual therapy 24%	Not reported	Not reported
Willeford (26)	Retrospective cohort	151	22/129	56	80	Mixed	Transthoracic echocardiography	Apixaban or rivaroxaban	Standard doses	Dual therapy 37%; triple therapy 19%	12 months	LVT resolution
Albabtain (27)	Retrospective cohort	63	28/35	59	91	Post–myocardial infarction	Transthoracic echocardiography	Rivaroxaban	20 mg once daily (reduced to 15 mg in some patients)	Dual antiplatelet therapy	254 days	Composite outcome
Zhang (28)	Retrospective cohort	64	33/31	60.8	73	ST-elevation myocardial infarction	Transthoracic echocardiography	Rivaroxaban	20 mg once daily	Triple therapy → dual therapy	25 months	LVT resolution
Liang (29)	Retrospective cohort	128	56/72	55.1	88	ST-elevation myocardial infarction	Transthoracic echocardiography	Rivaroxaban or dabigatran	Mixed dosing	Triple therapy common	12 months	LVT resolution
Herald (30)	Retrospective cohort	433	134/299	66	83	LVT	Transthoracic echocardiography	Multiple DOACs	Standard doses	P2Y12 inhibitor ∼45%	3.4 years	Ischemic composite
Zhou (28)	Retrospective cohort	240	111/129	55	88	Post–myocardial infarction	Transthoracic/transesophageal echocardiography	Rivaroxaban or dabigatran	Standard doses	Aspirin 42.3%; P2Y12 inhibitor 54.1%	1 year	LVT resolution
Al-Maimoony (31)	Retrospective cohort	302	183/119	Not reported	Not reported	LVT	Not reported	Apixaban or rivaroxaban	Dose adjusted	Aspirin 76.5%; clopidogrel 39.3%	12 months	LVT resolution
Paiva (32)	Retrospective cohort	171	99/72	59.8	83	LVT	Not reported	Multiple DOACs	Standard dosing	Triple therapy 14.6%	185 days	LVT resolution

Age is reported as mean or median as provided in the original studies.

Standard therapeutic DOAC dosing refers to apixaban 5 mg twice daily and rivaroxaban 20 mg once daily, unless otherwise specified. Dose adjustments were applied in selected studies based on clinical criteria.

Antiplatelet regimens were extracted when reported; NR indicates not reported.

Across the included studies, DOAC dosing was generally consistent with standard therapeutic regimens used for thromboembolic indications. Apixaban was most commonly administered at 5 mg twice daily, with dose reductions applied in selected patients, while rivaroxaban was typically prescribed at 20 mg once daily or 15 mg once daily in patients with renal impairment or increased bleeding risk.

Concomitant antiplatelet therapy was reported in several studies, particularly among post-MI and ACS populations. In these settings, dual antiplatelet therapy (DAPT) was commonly reported, and some studies described short-term triple antithrombotic therapy. However, detailed information regarding antiplatelet regimens and their duration was inconsistently reported across studies.

### Risk of bias in included studies

Overall, randomized controlled trials were judged to have low to some concerns of bias across most domains. In contrast, the majority of observational studies were rated as having moderate to serious risk of bias, primarily due to confounding and selection bias. Detailed risk-of-bias assessments for individual studies are presented in Supplementary Table S3A,B.

### LVT resolution

**Figure 2:**
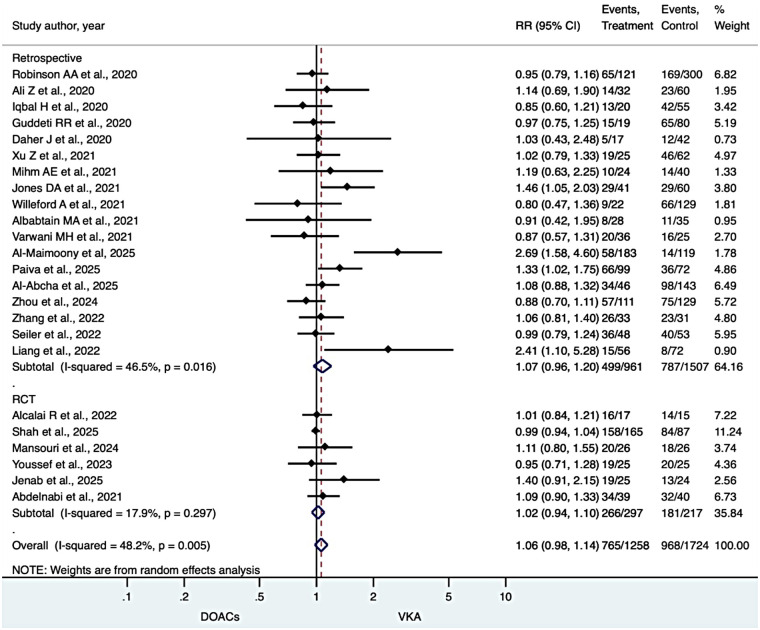
LVT resolution.

Across 28 included studies, DOAC therapy was associated with a numerically higher rate of LVT resolution compared with VKAs, although this did not reach statistical significance (overall RR 1.06, 95% CI 0.98–1.14), with moderate heterogeneity (I² = 48.2%). Subgroup analysis by study design demonstrated a consistent direction of effect in observational studies (RR 1.07, 95% CI 0.96–1.20; I² = 46.5%) and randomized controlled trials (RR 1.02, 95% CI 0.94–1.10; I² = 17.9%), with no significant interaction between subgroups. These findings suggest a modest improvement in thrombus resolution, although this did not reach statistical significance (Figure 2).

### Systemic embolic events

**Figure 3:**
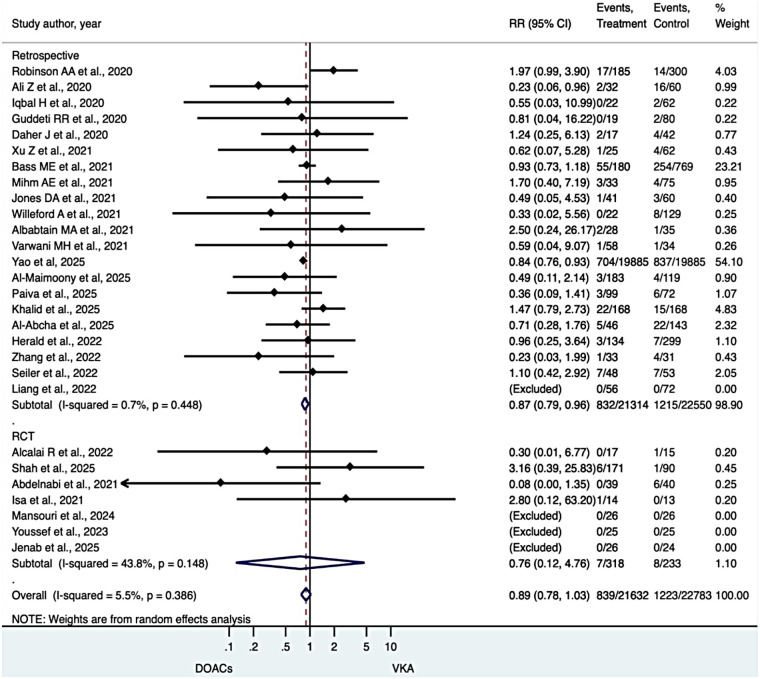
Systematic embolism.

No significant difference was observed between DOACs and VKAs in the risk of systemic embolic events. The pooled analysis demonstrated comparable outcomes between treatment groups (RR 0.89, 95% CI 0.78–1.03), with low heterogeneity (I² = 5.5%). Results were consistent across observational cohorts and randomized trials, indicating similar effectiveness of DOACs and VKAs in preventing embolic complications in patients with LVT (Figure 3).

### All-Cause mortality

**Figure 4:**
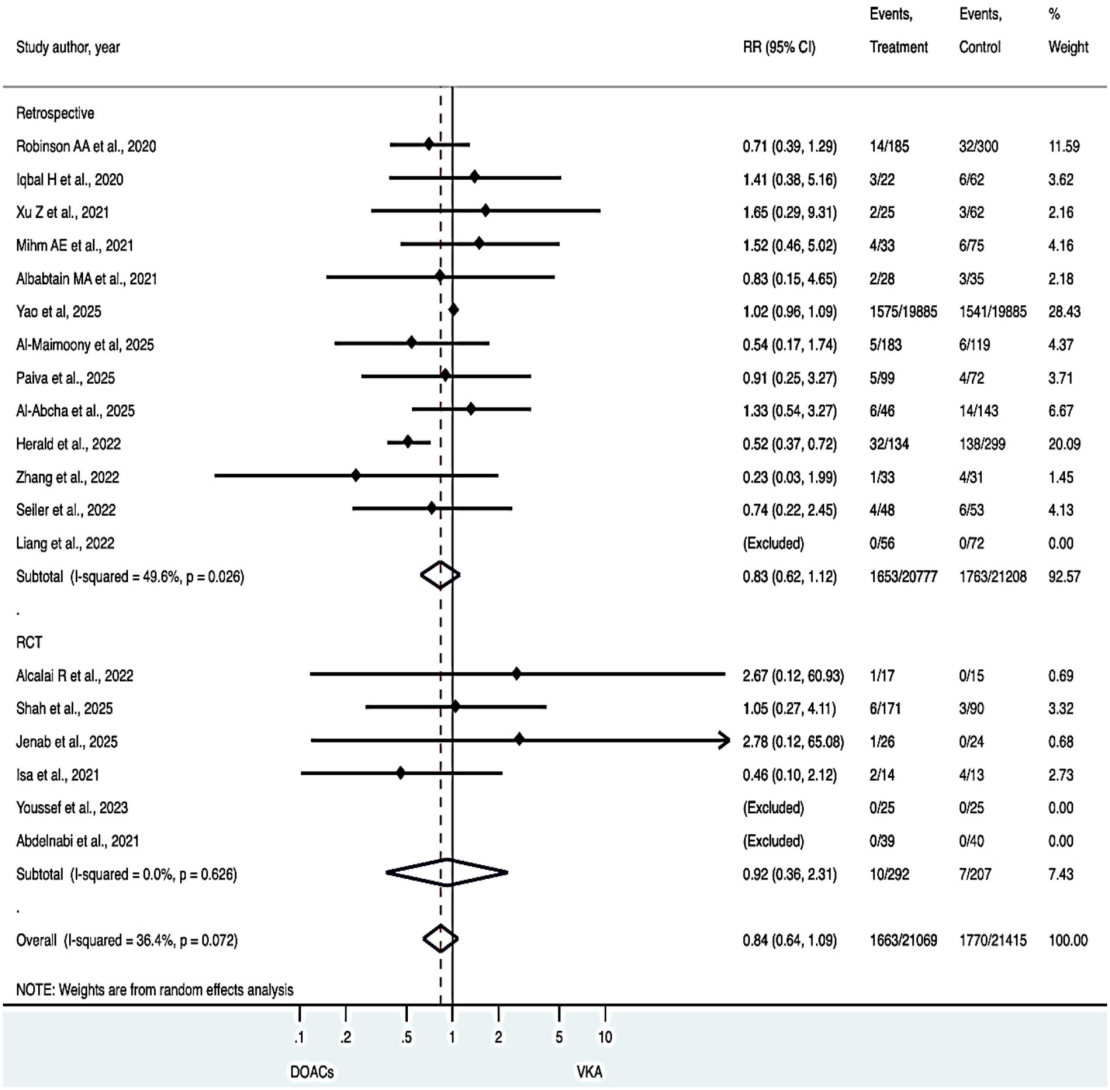
All cause-mortality.

DOAC use was associated with a numerically lower risk of all-cause mortality compared with VKAs, although this did not reach statistical significance (RR 0.84, 95% CI 0.64–1.09), with minimal heterogeneity (I² = 36.4%) (Figure 4).

### Bleeding outcomes

**Figure 5:**
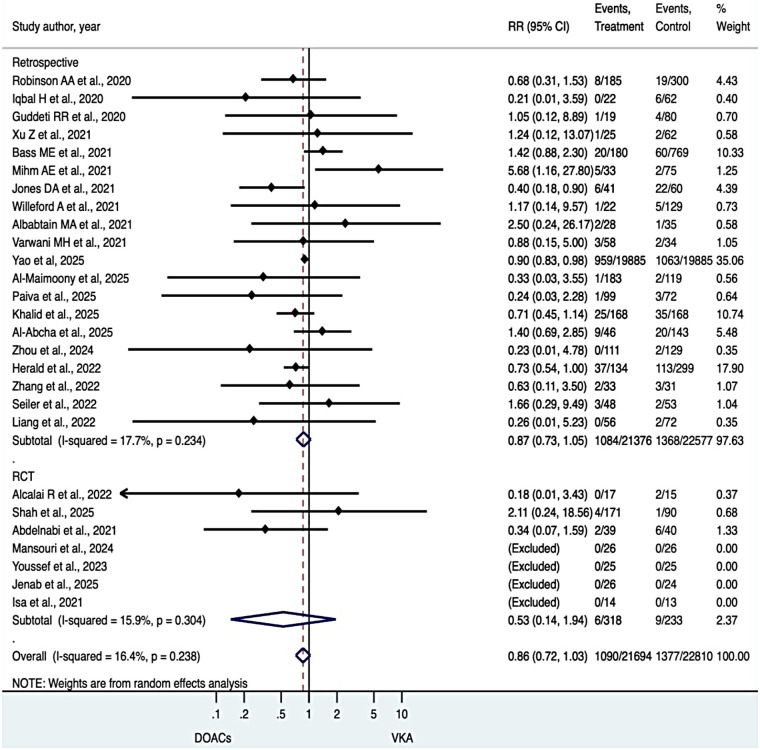
Bleeding outcomes.

Patients treated with DOACs had a numerically lower rate of bleeding events than those receiving VKAs, although this difference did not reach statistical significance (RR 0.86, 95% CI 0.72–1.03). Heterogeneity across studies was low to moderate (I² = 16.4%). These findings were directionally consistent across study designs (Figure 5).

## Discussion

This updated systematic review and meta-analysis synthesizes contemporary randomized and observational evidence comparing DOACs with VKAs for the treatment of LVT. By integrating data from 29 studies encompassing diverse clinical settings and LVT etiologies, the analysis provides a comprehensive assessment of thrombus resolution, embolic events, major bleeding outcomes, and all-cause mortality in routine practice. The primary findings indicate that DOAC therapy was associated with a numerically higher rate of LVT resolution compared with VKAs, although this did not reach statistical significance, along with numerically lower risks of bleeding and all-cause mortality, without an increased risk of systemic embolic events.

The observed trend toward improved thrombus resolution with DOACs suggests at least comparable antithrombotic effectiveness relative to warfarin in this setting. Importantly, this association remained directionally consistent across prespecified subgroup analyses, including randomized controlled trials and observational studies. This benefit was observed despite substantial heterogeneity in patient populations, imaging modalities, anticoagulant regimens, and follow-up durations. The consistency of effect direction across sensitivity analyses supports the robustness of the pooled estimates. At the same time, the absence of a significant difference in systemic embolic events between treatment strategies reinforces the clinical safety of DOACs for embolic prevention in LVT, a key concern that has historically limited their adoption.

When placed in the context of earlier literature, these findings align with and extend prior observational reports and mechanistic understanding of LVT formation following myocardial infarction, as well as contemporary clinical guidance on LVT management (1, 2). Historically, warfarin has been the standard therapy for LVT, supported primarily by mechanistic rationale and long-standing clinical experience rather than high-quality randomized evidence specific to this indication. Early observational studies suggested comparable thrombus resolution and embolic outcomes with DOACs, but concerns persisted regarding selection bias and residual confounding (32–38). More recent randomized trials, although limited by small sample sizes and low event rates, have demonstrated similar rates of LVT resolution and embolic events between DOACs, particularly rivaroxaban or apixaban, and warfarin, with trends toward improved safety profiles among DOAC-treated patients (13–18).

Given the limited number and size of randomized trials in this field, randomized and observational studies were pooled using random-effects models to provide an overall estimate of treatment effects, with prespecified subgroup and sensitivity analyses performed according to study design. The present meta-analysis builds on these data by incorporating newer studies and providing pooled estimates across both randomized and observational designs. The observed reduction in all-cause mortality associated with DOAC therapy is notable but should be interpreted cautiously, as this signal was driven largely by observational data and was not consistently observed in randomized trials. Residual confounding by indication, differences in baseline risk, and treatment selection cannot be excluded. In clinical practice, patients at higher risk, such as those who are older, frail, or have significant renal impairment, may be preferentially treated with VKAs rather than DOACs. This treatment selection pattern could result in a worse baseline prognosis in the VKA group and may partially explain the observed mortality difference. Nevertheless, the observed mortality signal remains biologically plausible, given the lower bleeding rates, fewer anticoagulation interruptions, and more predictable pharmacokinetics associated with DOAC use.

From a clinical perspective, these results are highly relevant to contemporary practice. Guideline recommendations for LVT management continue to favor VKAs largely by tradition, while acknowledging the growing off-label use of DOACs and the limited availability of high-certainty randomized evidence. Recent scientific statements and expert consensus documents increasingly recognize DOACs as reasonable alternatives to warfarin for LVT in selected patients, particularly when INR monitoring is challenging or bleeding risk is a concern (6–9). The findings of this meta-analysis provide quantitative support for this evolving practice pattern, suggesting that DOACs offer comparable effectiveness with a more favorable safety profile in appropriately selected patients.

The lower rates of bleeding associated with DOAC therapy are particularly important in LVT populations, where concomitant antiplatelet therapy following MI or percutaneous coronary intervention is common. In such settings, minimizing bleeding risk without compromising thrombus resolution or embolic protection is a key therapeutic goal. The absence of increased systemic embolic events in DOAC-treated patients in this analysis provides additional reassurance that simplified anticoagulation strategies may be safely implemented in suitable clinical contexts.

An important consideration in interpreting these findings is the variability in anticoagulant dosing and concomitant antiplatelet therapy across studies. Although most studies used standard therapeutic doses of DOACs, detailed reporting of dose adjustments and their relationship with clinical outcomes was limited. Concomitant antiplatelet therapy was reported in several studies, particularly among post-MI populations, with some describing the use of dual antiplatelet therapy and, in certain cases, short-term triple antithrombotic therapy. However, this information was inconsistently reported, and many studies did not provide detailed data on antiplatelet regimens or their duration. As a result, the independent impact of anticoagulant dosing and antiplatelet strategy on thrombus resolution and bleeding risk could not be reliably assessed. The overall consistency in thrombus resolution across studies suggests that treatment response may be driven more by patient-related factors, such as thrombus burden and left ventricular function, rather than modest variations in anticoagulant dosing or concomitant antithrombotic therapy. These findings highlight the need for future studies to better define optimal anticoagulation strategies, including dosing, duration, and integration with antiplatelet therapy in this high-risk population.

This study has several strengths. It represents one of the most comprehensive and up-to-date syntheses of evidence comparing DOACs and VKAs for LVT, incorporating both randomized and real-world data. The use of random-effects models, prespecified subgroup and sensitivity analyses, and formal risk-of-bias assessment enhances the credibility of the findings. Inclusion of diverse patient populations and clinical scenarios improves generalizability and reflects contemporary practice patterns.

Nevertheless, important limitations must be acknowledged. The overall evidence base remains dominated by observational studies, limiting causal inference and rendering the findings susceptible to confounding by indication and treatment selection bias. Imaging protocols and modalities varied substantially across studies, potentially influencing thrombus detection and resolution rates. Definitions of thrombus resolution, bleeding outcomes, anticoagulant dosing, and treatment duration were heterogeneous, and event adjudication was not uniform. Additionally, the modest effect sizes observed, particularly for thrombus resolution, should be interpreted within the context of clinical heterogeneity and limited randomized evidence. As such, while these findings support the use of DOACs as a viable alternative to warfarin, they do not obviate the need for adequately powered, LVT-specific randomized trials. Furthermore, detailed data on DOAC dosing strategies, duration of therapy after thrombus resolution, and concomitant antiplatelet regimens were inconsistently reported, limiting the ability to perform subgroup analyses based on these clinically relevant variables.

Despite these limitations, the present analysis provides meaningful real-world and trial-based evidence supporting the use of DOACs for LVT and contributes to the growing literature informing individualized anticoagulation strategies in this high-risk population.

## Conclusion

In this updated systematic review and meta-analysis, DOACs were associated with a numerically higher rate of LVT resolution, although this did not reach statistical significance, and a lower rate of bleeding compared with VKAs, without an increased risk of systemic embolic events. These findings suggest that DOACs represent an effective and safe alternative to warfarin for the management of LVT in routine clinical practice.

Although a reduction in rates of all-cause mortality was observed with DOAC therapy, this association was primarily driven by retrospective observational studies and should therefore be interpreted with caution, as residual confounding and treatment selection bias cannot be excluded. RCTs included in the analysis were underpowered to detect differences in mortality and did not demonstrate a consistent survival benefit.

Overall, the available evidence supports the use of DOACs as a reasonable and potentially safer alternative to VKAs in appropriately selected patients with LVT, particularly when bleeding risk or challenges with INR monitoring are present. However, adequately powered, LVT-specific randomized trials remain necessary to definitively establish comparative effectiveness and long-term clinical outcomes, including mortality.

## Data Availability

The original contributions presented in the study are included in the article/Supplementary Material, further inquiries can be directed to the corresponding author/s.
