# Supplementary material for: Direct oral anticoagulants versus warfarin for left ventricular thrombus: an updated systematic review and meta-analysis of randomized and observational studies
Source: Front Cardiovasc Med. 2026 May 14;13:1814694. doi: 10.3389/fcvm.2026.1814694 (PMC13216051; doi:10.3389/fcvm.2026.1814694)
Supplement: Supplementary file 1 [file Supplementaryfile1.docx]

**Supplementary Materials**

**Supplement Table S1.** PRISMA 2020 Checklist

| **Section and Topic** | **Item #** | **Checklist item** | **Location where item is reported** |
| --- | --- | --- | --- |
| **TITLE** | | |  |
| Title | 1 | Identify the report as a systematic review. | Title page |
| **ABSTRACT** | | |  |
| Abstract | 2 | See the PRISMA 2020 for Abstracts checklist. | Abstract |
| **INTRODUCTION** | | |  |
| Rationale | 3 | Describe the rationale for the review in the context of existing knowledge. | Introduction |
| Objectives | 4 | Provide an explicit statement of the objective(s) or question(s) the review addresses. | Introduction (last paragraph) |
| **METHODS** | | |  |
| Eligibility criteria | 5 | Specify the inclusion and exclusion criteria for the review and how studies were grouped for the syntheses. | Methods – Eligibility Criteria |
| Information sources | 6 | Specify all databases, registers, websites, organisations, reference lists and other sources searched or consulted to identify studies. Specify the date when each source was last searched or consulted. | Methods – Information Sources |
| Search strategy | 7 | Present the full search strategies for all databases, registers and websites, including any filters and limits used. | Supplementary Methods; Supplementary Table S2 |
| Selection process | 8 | Specify the methods used to decide whether a study met the inclusion criteria of the review, including how many reviewers screened each record and each report retrieved, whether they worked independently, and if applicable, details of automation tools used in the process. | Methods – Study Selection |
| Data collection process | 9 | Specify the methods used to collect data from reports, including how many reviewers collected data from each report, whether they worked independently, any processes for obtaining or confirming data from study investigators, and if applicable, details of automation tools used in the process. | Methods – Data Extraction |
| Data items | 10a | List and define all outcomes for which data were sought. Specify whether all results that were compatible with each outcome domain in each study were sought (e.g. for all measures, time points, analyses), and if not, the methods used to decide which results to collect. | Methods – Outcomes |
|  | 10b | List and define all other variables for which data were sought (e.g. participant and intervention characteristics, funding sources). Describe any assumptions made about any missing or unclear information. | Methods – Data Items |
| Study risk of bias assessment | 11 | Specify the methods used to assess risk of bias in the included studies, including details of the tool(s) used, how many reviewers assessed each study and whether they worked independently, and if applicable, details of automation tools used in the process. | Methods – Risk of Bias Assessment |
| Effect measures | 12 | Specify for each outcome the effect measure(s) (e.g. risk ratio, mean difference) used in the synthesis or presentation of results. | Methods – Statistical Analysis |
| Synthesis methods | 13a | Describe the processes used to decide which studies were eligible for each synthesis (e.g. tabulating the study intervention characteristics and comparing against the planned groups for each synthesis (item #5)). | Methods – Data Synthesis |
|  | 13b | Describe any methods required to prepare the data for presentation or synthesis, such as handling of missing summary statistics, or data conversions. | Methods – Data Synthesis |
|  | 13c | Describe any methods used to tabulate or visually display results of individual studies and syntheses. | Methods – Data Synthesis; Figures 2–6 |
|  | 13d | Describe any methods used to synthesize results and provide a rationale for the choice(s). If meta-analysis was performed, describe the model(s), method(s) to identify the presence and extent of statistical heterogeneity, and software package(s) used. | Methods – Data Synthesis and Statistical Analysis |
|  | 13e | Describe any methods used to explore possible causes of heterogeneity among study results (e.g. subgroup analysis, meta-regression). | Methods – Subgroup and Sensitivity Analyses |
|  | 13f | Describe any sensitivity analyses conducted to assess robustness of the synthesized results. | Methods – Subgroup and Sensitivity Analyses |
| Reporting bias assessment | 14 | Describe any methods used to assess risk of bias due to missing results in a synthesis (arising from reporting biases). | Methods – Publication Bias |
| Certainty assessment | 15 | Describe any methods used to assess certainty (or confidence) in the body of evidence for an outcome. | Not assessed |
| **RESULTS** | | |  |
| Study selection | 16a | Describe the results of the search and selection process, from the number of records identified in the search to the number of studies included in the review, ideally using a flow diagram. | Results – Study Selection; Figure 1 (PRISMA) |
|  | 16b | Cite studies that might appear to meet the inclusion criteria, but which were excluded, and explain why they were excluded. | Results – Study Selection; Figure 1 |
| Study characteristics | 17 | Cite each included study and present its characteristics. | Results – Study Characteristics; Table 1 |
| Risk of bias in studies | 18 | Present assessments of risk of bias for each included study. | Results – Risk of Bias; Tables S3 A and B |
| Results of individual studies | 19 | For all outcomes, present, for each study: (a) summary statistics for each group (where appropriate) and (b) an effect estimate and its precision (e.g. confidence/credible interval), ideally using structured tables or plots. | Results; Figures 2–6; Supplementary Tables |
| Results of syntheses | 20a | For each synthesis, briefly summarise the characteristics and risk of bias among contributing studies. | Results – Meta-analysis |
|  | 20b | Present results of all statistical syntheses conducted. If meta-analysis was done, present for each the summary estimate and its precision (e.g. confidence/credible interval) and measures of statistical heterogeneity. If comparing groups, describe the direction of the effect. | Results – Meta-analysis; Figures 2–6 |
|  | 20c | Present results of all investigations of possible causes of heterogeneity among study results. | Results – Subgroup Analyses |
|  | 20d | Present results of all sensitivity analyses conducted to assess the robustness of the synthesized results. | Results – Sensitivity Analyses |
| Reporting biases | 21 | Present assessments of risk of bias due to missing results (arising from reporting biases) for each synthesis assessed. | Results – Publication Bias; Figures S1–S4 |
| Certainty of evidence | 22 | Present assessments of certainty (or confidence) in the body of evidence for each outcome assessed. | Not assessed |
| **DISCUSSION** | | |  |
| Discussion | 23a | Provide a general interpretation of the results in the context of other evidence. | Discussion |
|  | 23b | Discuss any limitations of the evidence included in the review. | Discussion – Limitations |
|  | 23c | Discuss any limitations of the review processes used. | Discussion – Limitations |
|  | 23d | Discuss implications of the results for practice, policy, and future research. | Discussion – Clinical Implications |
| **OTHER INFORMATION** | | |  |
| Registration and protocol | 24a | Provide registration information for the review, including register name and registration number, or state that the review was not registered. | Methods; PROSPERO CRD420251181379 |
|  | 24b | Indicate where the review protocol can be accessed, or state that a protocol was not prepared. | Methods |
|  | 24c | Describe and explain any amendments to information provided at registration or in the protocol. | Not applicable |
| Support | 25 | Describe sources of financial or non-financial support for the review, and the role of the funders or sponsors in the review. | Funding Statement |
| Competing interests | 26 | Declare any competing interests of review authors. | Conflicts of Interest |
| Availability of data, code and other materials | 27 | Report which of the following are publicly available and where they can be found: template data collection forms; data extracted from included studies; data used for all analyses; analytic code; any other materials used in the review. | Data Availability Statement |

*From:*  Page MJ, McKenzie JE, Bossuyt PM, Boutron I, Hoffmann TC, Mulrow CD, et al. The PRISMA 2020 statement: an updated guideline for reporting systematic reviews. BMJ 2021;372:n71. doi: 10.1136/bmj.n71

**Table S2.** Full electronic search strategies

| **Database** | **Search strategy** |
| --- | --- |
| **PubMed / MEDLINE** | ( "Left Ventricular Thrombus"[tiab] OR "Left Ventricular Thrombi"[tiab] OR "left ventricular thromb*"[tiab] OR "ventricular thrombus"[tiab] OR "intracardiac thrombus"[tiab] OR LVT[tiab] OR ( ( "Heart Ventricles"[Mesh] OR left ventric*[tiab] OR "Ventricular Function, Left"[Mesh] ) AND ( "Thrombosis"[Mesh] OR thrombos*[tiab] OR "blood clot*"[tiab] ) ) ) AND ( "Warfarin"[Mesh] OR warfarin[tiab] OR "Vitamin K Antagonists"[Mesh] OR VKA*[tiab] OR "vitamin K antagonist*"[tiab] ) AND ( "Factor Xa Inhibitors"[Mesh] OR "Thrombin Inhibitors"[Mesh] OR "direct oral anticoagulant*"[tiab] OR DOAC*[tiab] OR NOAC*[tiab] OR apixaban[tiab] OR rivaroxaban[tiab] OR edoxaban[tiab] OR dabigatran[tiab] ) NOT ( case reports[Publication Type] OR "case report"[tiab] ) NOT ( animals[MeSH Terms] NOT humans[MeSH Terms] ) |
| **Embase (Elsevier)** | 1. ‘left ventricle thrombosis’/exp OR ‘ventricle thrombosis’/exp OR (‘left ventricular thrombus’:ti,ab,kw OR ‘left ventricular thromb*’:ti,ab,kw OR ‘ventricular thrombus’:ti,ab,kw OR ‘intracardiac thrombus’:ti,ab,kw OR lvt:ti,ab,kw) 2. ‘warfarin’/exp OR warfarin:ti,ab,kw OR ‘vitamin k antagonist’/exp OR vka*:ti,ab,kw OR ‘vitamin k antagonist*’:ti,ab,kw 3. ‘direct oral anticoagulant’/exp OR ‘factor xa inhibitor’/exp OR ‘thrombin inhibitor’/exp OR (‘direct oral anticoagulant*’:ti,ab,kw OR doac*:ti,ab,kw OR noac*:ti,ab,kw OR apixaban:ti,ab,kw OR rivaroxaban:ti,ab,kw OR edoxaban:ti,ab,kw OR dabigatran:ti,ab,kw) 4. 1 AND 2 AND 3 5. 4 NOT (‘case report’/de) 6. 5 AND ‘human’/de |
| **Cochrane CENTRAL** | #1 ( “left ventricular thrombus” OR “left ventricular thromb*” OR LVT OR “ventricular thrombus” OR “intracardiac thrombus” ) #2 ( warfarin OR “vitamin k antagonist*” OR VKA* ) #3 ( “direct oral anticoagulant*” OR DOAC* OR NOAC* OR apixaban OR rivaroxaban OR edoxaban OR dabigatran ) #4 #1 AND #2 AND #3 |

**Table 3S.** Risk of Bias Assessment of Included Studies

**A. Randomized Controlled Trials (RoB 2.0)**

| **Study (First author, year)** | **Randomization process** | **Deviations from intended interventions** | **Missing outcome data** | **Measurement of outcomes** | **Selection of reported results** | **Overall risk of bias** |
| --- | --- | --- | --- | --- | --- | --- |
| Alcalai 2022 | \| Low risk \| \| --- \| | Some concerns | Low risk | Low risk | Low risk | Some concerns |
| Youssef 2023 | Low risk | Low risk | Low risk | Low risk | Low risk | Low risk |
| Jenab 2025 | Low risk | Low risk | Low risk | Low risk | Low risk | Low risk |
| Mansouri 2024 | Low risk | Some concerns | Low risk | Some concerns | Low risk | Some concerns |
| Shah 2025 | Low risk | High risk | High risk | Low risk | Low risk | High risk |
| Isa 2021 | Low risk | Some concerns | High risk | Low risk | Some concerns | High risk |
| Abdelnabi 2021 | Some concerns | Some concerns | Low risk | High risk | Low risk | High risk |

**B. Observational Studies (ROBINS-I)**

| **Study (First author, year)** | **Confounding** | **Participant Selection** | **Intervention Classification** | **Deviations from Intended Interventions** | **Missing Data** | **Outcome Measurement** | **Selective Reporting** | **Overall Risk** |
| --- | --- | --- | --- | --- | --- | --- | --- | --- |
| **Robinson, 2020** | Serious | Serious | Moderate | Serious | Moderate | Moderate | Moderate | **Serious** |
| **Ali, 2020** | Serious | Serious | Moderate | Serious | Moderate | Moderate | Moderate | **Serious** |
| **Iqbal, 2020** | Serious | Serious | Moderate | Serious | Moderate | Moderate | Moderate | **Serious** |
| **Guddeti, 2020** | Serious | Serious | Moderate | Serious | Moderate | Moderate | Moderate | **Serious** |
| **Daher, 2020** | Serious | Serious | Moderate | Serious | Moderate | Moderate | Moderate | **Serious** |
| **Bass, 2021** | Serious | Serious | Moderate | Serious | Moderate | Moderate | Moderate | **Serious** |
| **Mihm, 2021** | Serious | Serious | Moderate | Serious | Serious | Moderate | Moderate | **Serious** |
| **Varwani, 2021** | Serious | Moderate | Moderate | Serious | Serious | Moderate | Moderate | **Serious** |
| **Yao, 2025** | Serious | Moderate | Moderate | Serious | Moderate | Moderate | Moderate | **Serious** |
| **Al-Maimoony, 2025** | Moderate | Serious | Low | Moderate | Serious | Low | Moderate | **Serious** |
| **Khalid, 2025** | Moderate | Moderate | Moderate | Serious | Moderate | Moderate | Moderate | **Serious** |
| **Zhou, 2024** | Serious | Moderate | Moderate | Moderate | Low | Moderate | Moderate | **Serious** |
| **Xu, 2021** | Moderate | Moderate | Low | Moderate | Low | Moderate | Moderate | **Moderate** |
| **Jones, 2021** | Moderate | Moderate | Low | Moderate | Low | Low | Moderate | **Moderate** |
| **Willeford, 2021** | Moderate | Moderate | Low | Moderate | Moderate | Moderate | Moderate | **Moderate** |
| **Albabtain, 2021** | Moderate | Moderate | Low | Moderate | Low | Moderate | Moderate | **Moderate** |
| **Paiva, 2025** | Moderate | Moderate | Low | Moderate | Low | Low | Moderate | **Moderate** |
| **Al-Abcha, 2025** | Moderate | Moderate | Moderate | Moderate | Moderate | Moderate | Moderate | **Moderate** |
| **Herald, 2022** | Moderate | Moderate | Moderate | Moderate | Moderate | Moderate | Moderate | **Moderate** |
| **Zhang, 2022** | Moderate | Moderate | Low | Moderate | Moderate | Moderate | Moderate | **Moderate** |
| **Seiler, 2022** | Moderate | Moderate | Moderate | Moderate | Moderate | Moderate | Moderate | **Moderate** |
| **Liang, 2022** | Moderate | Moderate | Low | Moderate | Low | Moderate | Moderate | **Moderate** |

**
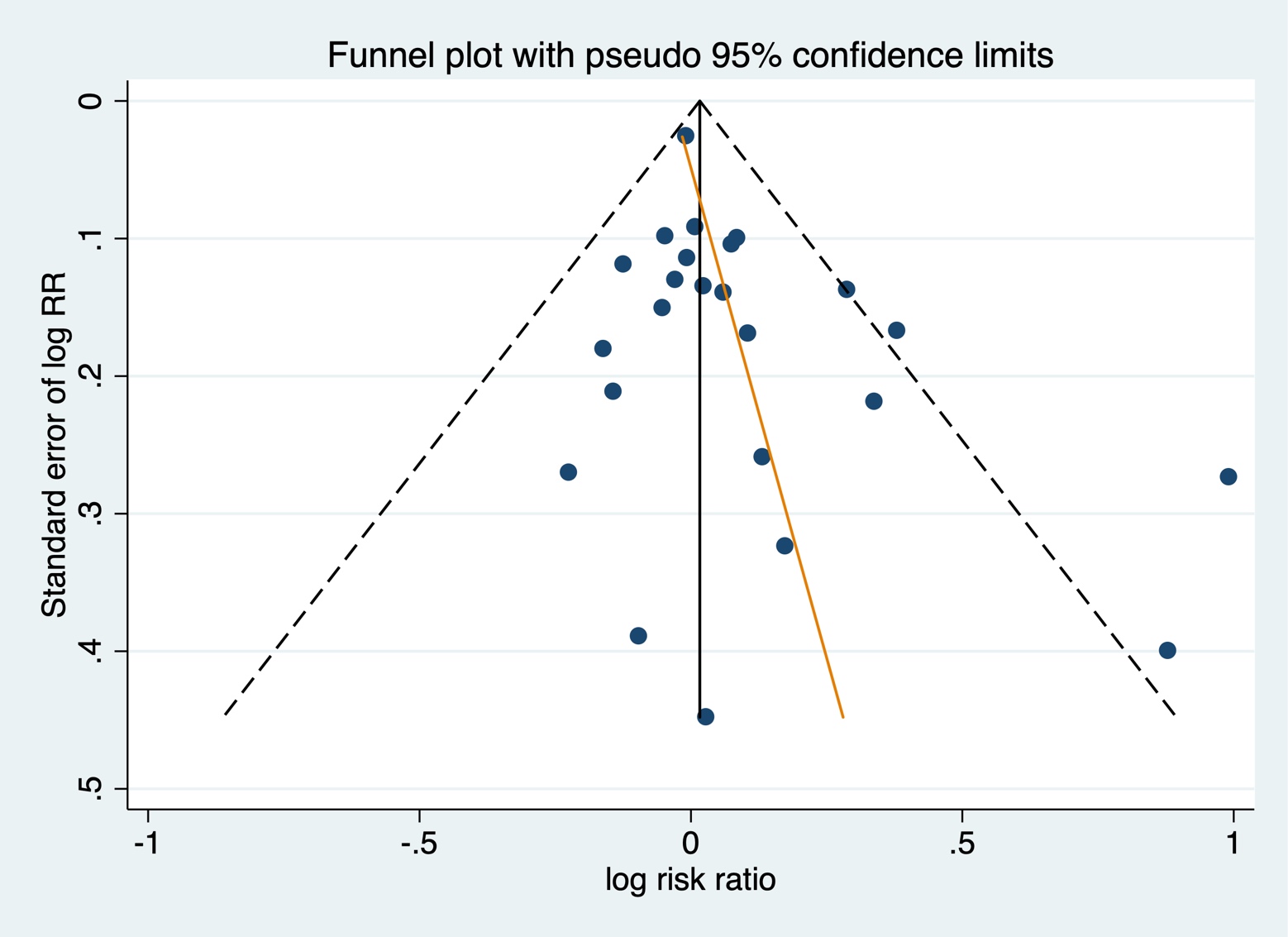
**

**Figure S1. Funnel plot for LVT**


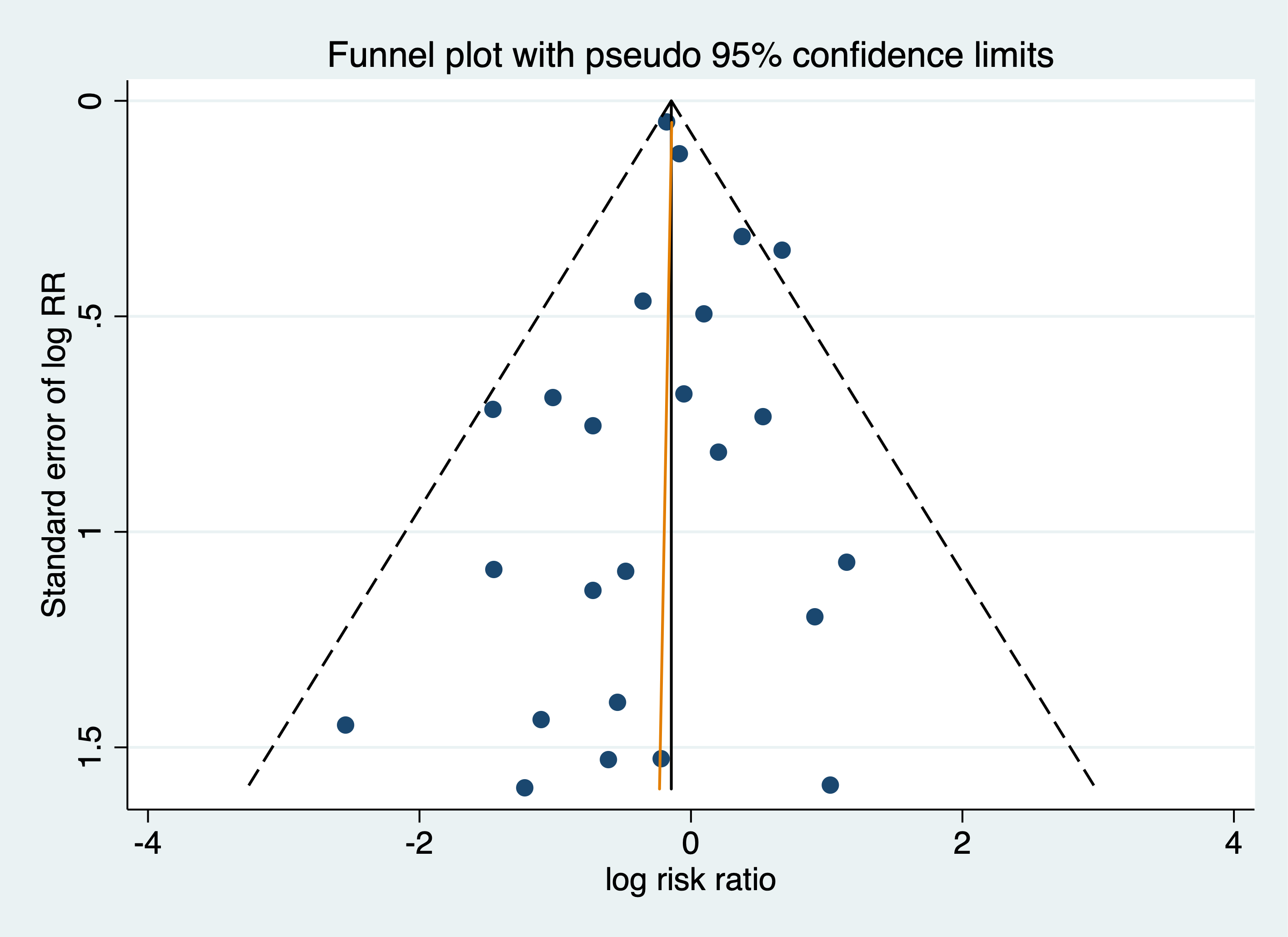


**Figure S2. Funnel plot for Systematic Embolism**

**
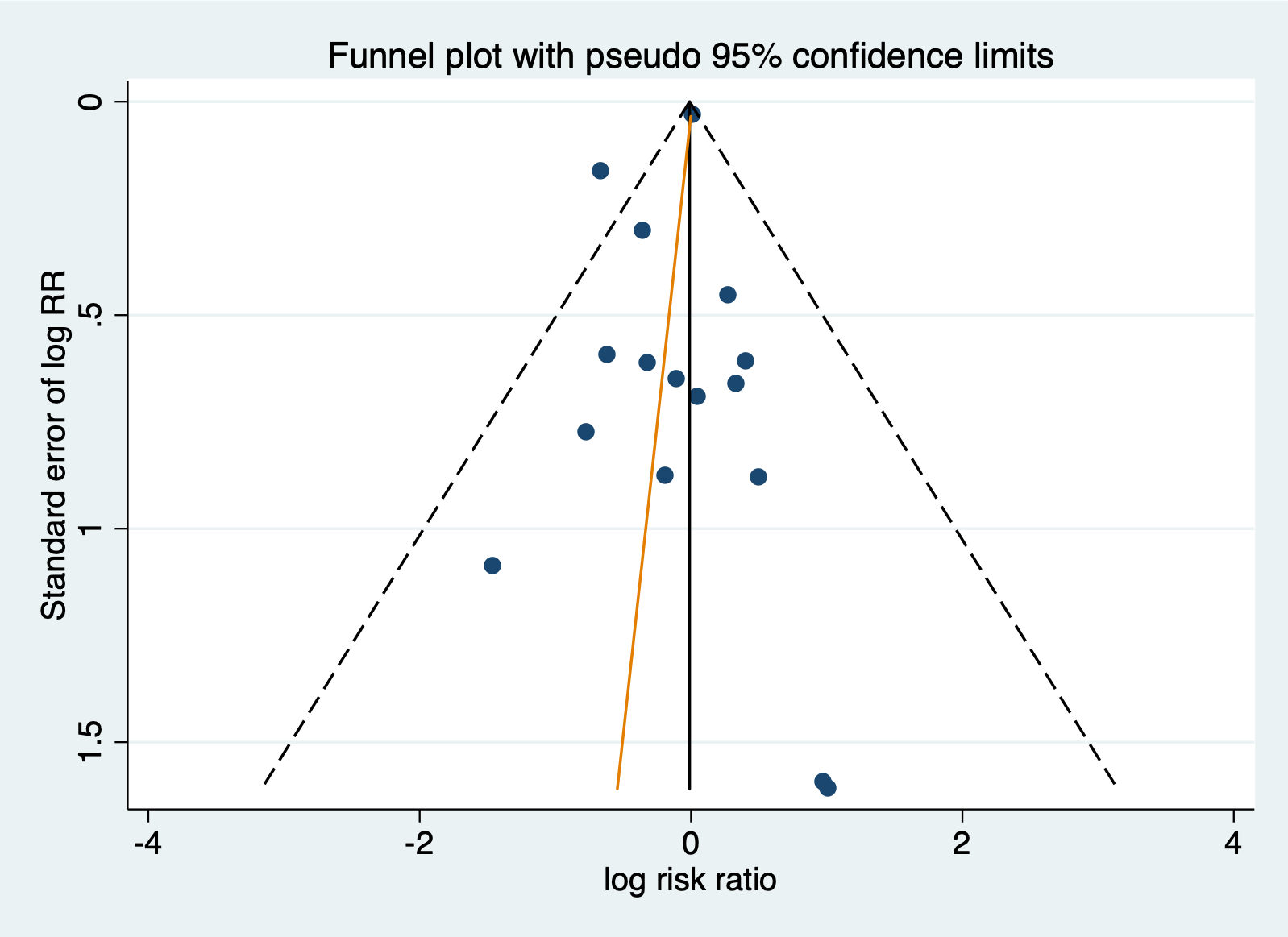
**

**Figure S4. Funnel plot for Mortality**

**
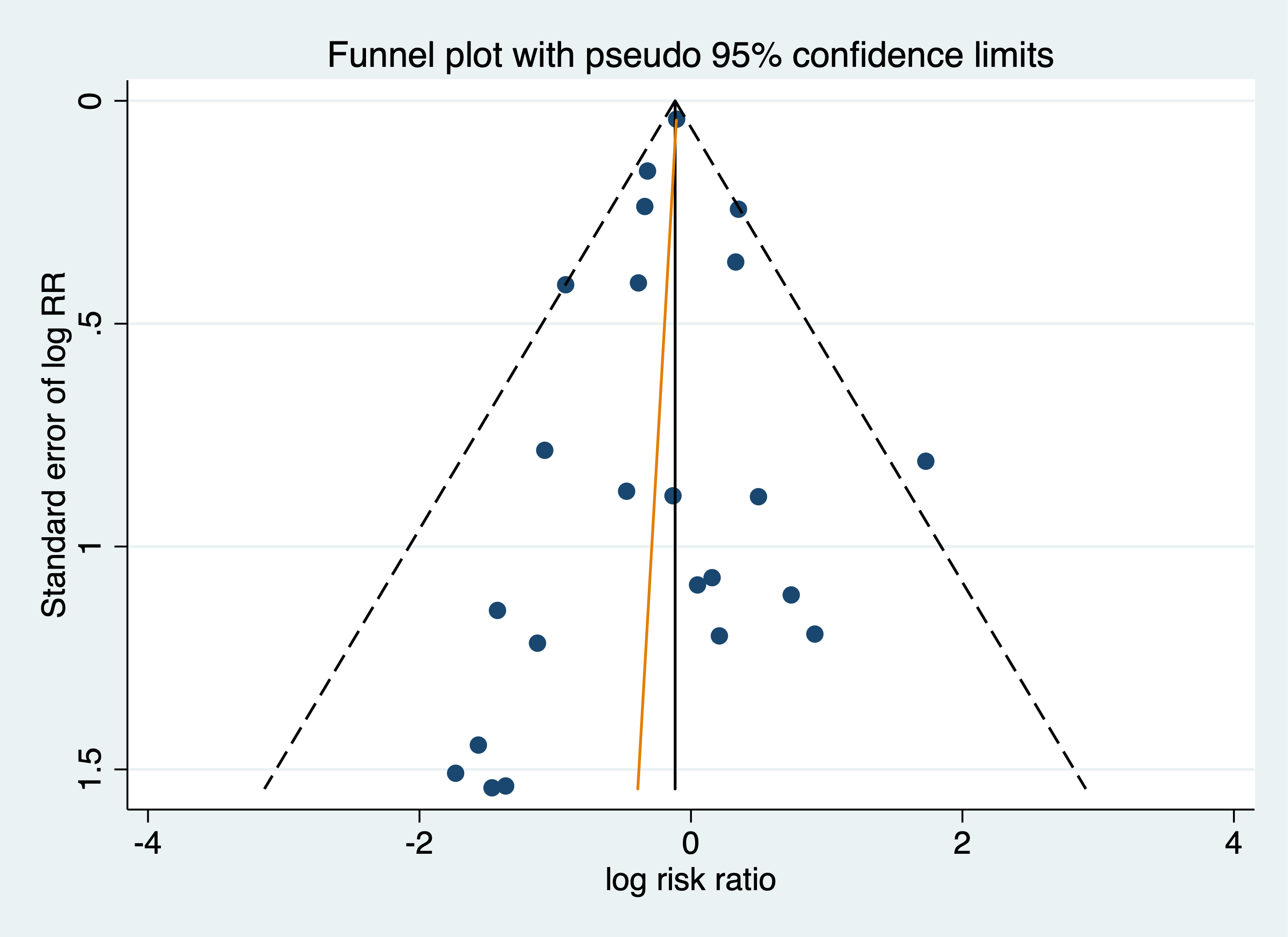
**

**Figure S3. Funnel plot for Bleeding**
